# Concentrations of Atmospheric Sulfur Compounds in an Extremely Snowy Region, the Hokuriku District, Japan

**DOI:** 10.1100/tsw.2004.23

**Published:** 2004-03-26

**Authors:** Tomonori Kawakami, Jun Murayama

**Affiliations:** Toyama Prefectural University, Department of Environmental Technology, 5180 Kurokawa, Kosugi, Toyama 939-0398, Japan

**Keywords:** sulfur dioxide, sulfate, diurnal pattern, long-range transportation, cloud base, washout ratio

## Abstract

The diurnal and seasonal characteristics in gaseous sulfur dioxide and sulfate in aerosol particles, as well as the concentrations of sulfate in rain and snow, were measured in the Hokuriku District, Japan in order to investigate the spatial spread pattern of sulfur compounds and identify the origin of sulfur. The concentration of sulfur dioxide showed a distinct diurnal pattern, while the concentrations of nss-SO_4_ in precipitation and aerosol particles did not. These results implied that the sulfur dioxide might originate in local emissions and did not affect the concentration of nss-SO_4_ in precipitation, while nss-SO_4_ in aerosol particles seemed to be widespread and might result from long-range transportation. The deposition of nss-SO_4_ in precipitation increased in winter, while the concentration of nss-SO_4_ in aerosol particles decreased. This could be attributed to the lower cloud base often observed in this district in winter associated with a higher washout ratio.

### INTRODUCTION

It is well documented that a more elevated wet sulfate deposition and a more decreased pH are observed in winter than in summer in the part of the Hokuriku District that faces the Japan Sea[1]. This is believed to be due to the extremely high volume of snowfall that contains a high concentration of sulfate[2,3]. Because the typical pattern of air pressure in winter, i.e., high in the west and low in the east, causes northwestern wind over Japan, which brings much snowfall to the district, it is likely that the air that travels across the Japan Sea contains the causal substances of a high concentration of sulfate in snow. There is evidence of long-range transportation of air pollutants from the Asian Continent. Kitamura et al.[4] investigated the sulfur isotope ratio in snow deposited in this district to conclude that the sulfate was transported from the northern part of the Chang Jiang River. Mukai et al.[5] found that lead isotope ratios in snow samples collected in Japan were similar to those of the leads yielded in Russia and China. However, there are still controversial arguments about the origins of the sulfate in snow and rain in winter, that is, whether the high concentration of sulfate is brought by long-range transportation or by local emission. Murao et al.[6] estimated, using a numerical simulation, that about 90% of the sulfate deposited in February 1998 in Toyama City in the Hokuriku District had a foreign origin. Ikeda et al.[7] also used a numerical model to estimate dry and wet deposition in Japan and reported that about 70% of the sulfur deposited in areas around the Japan Sea in winter was attributed to a foreign origin. On the other hand, Honoki and Hayakawa[8] used an empirical model, in which concentrations of pollutants contained in precipitation were assumed to decrease exponentially along with the distance from the coastline, and compared the estimated and observed concentrations of nonsea-salt sulfate to estimate that 71% of sulfate had a domestic origin in this district. Ohizumi et al.[9] suggested that only 20% of the sulfate deposited in Nagaoka City in the Hokuriku District originated in the Asian Continent. In this study, we measured diurnal and seasonal characteristics in gaseous sulfur dioxide and sulfate in aerosol particles, as well as the concentrations of sulfate in rain and snow, in order to investigate the spatial spread pattern of sulfur compounds and identify the origin of sulfur.

### METHODS

The concentrations of gaseous sulfur dioxide, sulfate concentrations in precipitation, and atmospheric concentration of aerosol sulfate were measured in order to assess the diurnal and seasonal changes in the concentrations of these sulfur compounds. All the measurements were performed in Kosugi, a town (36°44’N 137°7’E ) near Toyama City, Hokuriku District, Japan, from November 1999 to July 2001. The annual average precipitation in Kosugi amounts to about 2,250 mm, of which 800 mm are brought in winter from December to March. The depth of snow sometimes reaches 60 cm or more; however, snowmelt occurs even in midwinter except in the mountainous areas.

The sulfur dioxide concentration was monitored continuously by a sulfur dioxide analyzer using ultraviolet fluorescence (HORIBA APSA-365) and recorded every 10 min. The detection limit of this model was as low as 0.1 ppbv. To carry out an accurate zero calibration for the sulfur dioxide analyzer, pure air (SUMITOMO SEIKA CHEMICAL Zero-u) was used as a source gas for a calibration gas generator (STEC SGGU-514). The purified gas from the calibration gas generator was led to an activated carbon column before it was introduced into the sulfur dioxide analyzer.

Precipitation was collected by a funnel collector with a diameter of 20 cm. In winter, snow was collected after it was melted with a temperature-controlled heater inside the funnel. To collect daytime and nighttime precipitation separately, two plastic bottles were placed under the funnel collector, and the flow path from the funnel to the inlets of the two bottles was switched alternatively. The changeover times of daytime and nighttime were 6 a.m. and 8 p.m., according to the electric consumption data supplied by a local electric power company. The precipitation samples were collected weekly. Major ions were analyzed by ion chromatography (YOKOGAWA IC-7000) after filtration with membrane filters.

Daytime and nighttime aerosol particles were also collected separately on quartz filters by using a high-volume air sampler (SHIBATA HVC-1000N) with a flow rate of 1,000 l min$^{-1}$. Water-soluble components were extracted by an ultrasonic method from the quartz filters. Major ions were analyzed by ion chromatography after filtration with membrane filters. In this experiment, the nighttime sampling was done consecutively after daytime sampling; however, because we needed several hours to exchange filters, daytime sampling was done from 10 a.m. to 6 p.m., while nighttime sampling was done from 10 p.m. to 6 a.m. To avoid the effect of washout by rain and snow on the concentration of aerosol sulfate, the air pump in the high-volume air sampler was stopped when the rain sensor detected rain and snow. Aerosol particles were sampled more than once a month when it was not raining. On the contrary, aerosol particles were sometimes collected while it was raining in order to estimate the washout ratio by operating the pump only when the rain sensor detected rain and snow.

## RESULTS AND DISCUSSION

### Sulfur Dioxide

The typical diurnal patterns of atmospheric concentrations of gaseous sulfur dioxide in winter and summer are shown in Figs. 1(a) and 1(b), respectively. A distinct diurnal cycle, in which the concentration was around 1 ppb in the daytime, but decreased below the detection limit of 0.1 ppb in the nighttime, was found in both seasons. These cycles have been observed for more than 4 years from 1999 regardless of the season. Feliciano et al.[10] observed higher deposition velocities in daytime than nighttime over short vegetation in Portugal. Xu and Carmichael[11] estimated by their model that the deposition velocities of sulfur dioxide were higher in the daytime than in the nighttime in Asia because of the decrease of stomata resistance in the daytime. Although the higher deposition velocities of sulfur dioxide in the daytime were expected, the concentration of sulfur dioxide in the nighttime was much lower than that in the daytime. In addition, the quick change in the concentration of sulfur dioxide illustrated in Figs. 1(a) and 1(b) supported the inference that the sources of sulfur dioxide could be attributed to local emissions in this area. The major local sources of sulfur dioxide in Kosugi are a coal-fired power plant and some chemical and petrochemical plants located several kilometers north of the town along the coast.

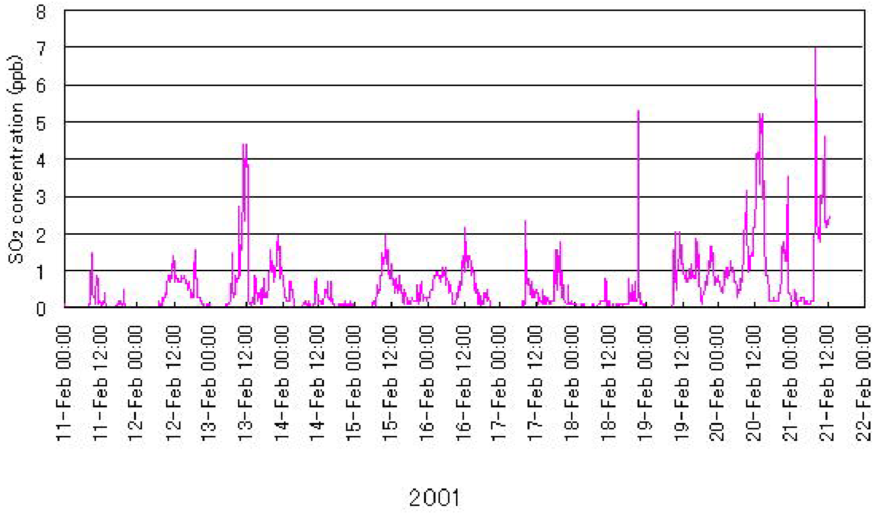

**FIGURE 1(a).** Diurnal change in $SO_2$ gas concentration in the winter.

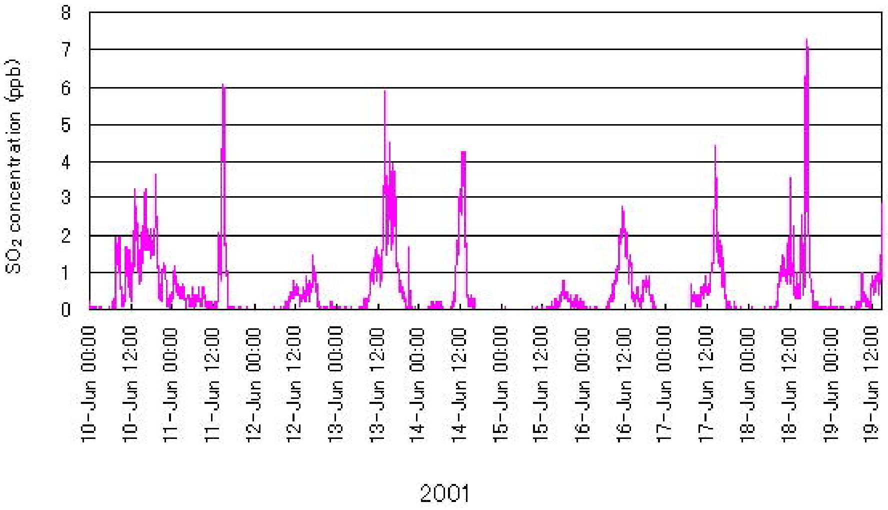

**FIGURE 1(b).** Diurnal change in $SO_2$ gas concentration in the summer.

Lee et al.[12] reported a distinct seasonal pattern, in which the concentration of sulfur dioxide in South Korea was higher in winter than in other seasons. Sulfur dioxide in South Korea, of which concentration reached 20 ppbv in winter, did not seem to affect the concentration in Kosugi, because no seasonal pattern in the concentration of sulfur dioxide was seen.

### Precipitation

Wet deposition of nonsea-salt sulfate (nss-$SO_4^{2-}$) is shown in Fig. 2. Elevated nss-$SO_4^{2-}$ deposition was observed in the winter as in previous reports[2,13]. A large amount of precipitation of 847 mm from December 2000 to March 2001 contributed to the elevated nss-$SO_4^{2-}$ deposition. The nss-$SO_4^{2-}$ concentrations in daytime and nighttime precipitation are illustrated in Fig. 3. Unlike sulfur dioxide, there

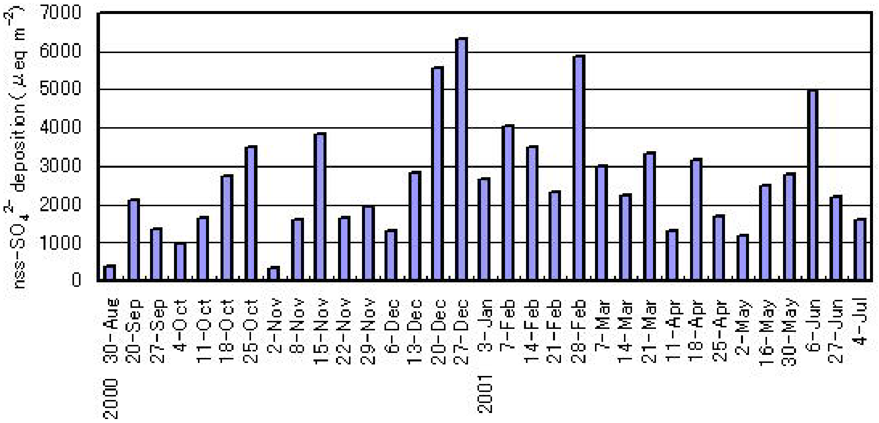

**FIGURE 2.** Wet deposition of nss-$SO_4^{2-}$ from August 2000 to July 2001.

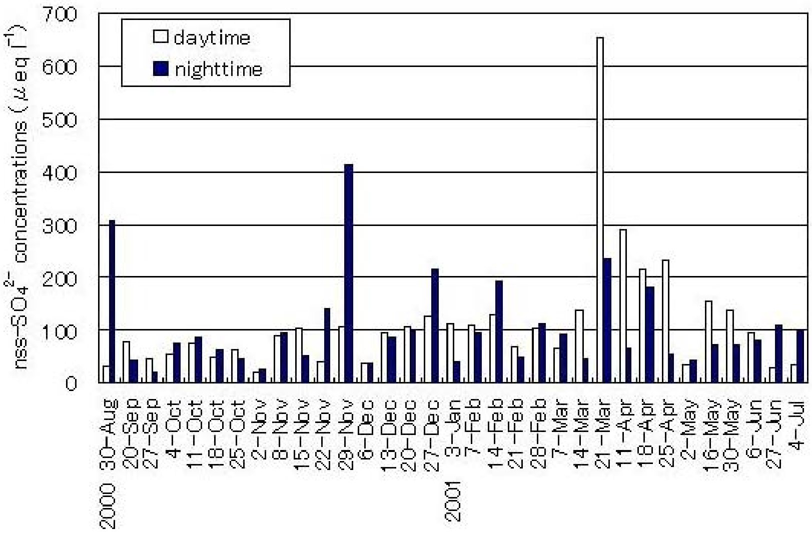

**FIGURE 3**. nss-$SO_4^{2-}$ concentrations in the daytime and nighttime precipitation.

was not a distinct pattern in the nss-$SO_4^{2-}$ concentrations between daytime and nighttime. The weighted average concentrations of nss-$SO_4^{2-}$ in precipitation, which were 111 and 102 µeq/l for daytime and nighttime, respectively, were almost identical. The concentration of nss-$SO_4^{2-}$ in precipitation without a diurnal pattern indicated that it was not affected directly by the atmospheric concentration of sulfur dioxide that was measured at ground level but by other controlling factors.

The nss-$SO_4^{2-}$ concentrations did not seem to increase, even in midwinter. Fujita et al.[14] conducted a wet deposition monitoring at six rural stations in western Japan, and found a clear seasonal pattern in the concentration of nss-$SO_4^{2-}$. This difference may be due to the larger distance from the Asian Continent to the Hokuriku District than to Fujita's stations.

### Aerosol Particles

The atmospheric concentrations of nss-$SO_4^{2-}$ in aerosol particles in the daytime and nighttime are shown in Fig. 4. Unlike sulfur dioxide, the change in the concentrations from the daytime to the nighttime of nss-$SO_4^{2-}$ in aerosol particles was not significant throughout the year. This indicated that nss-$SO_4^{2-}$ in aerosol particles was widespread and could be attributed to long-range transportation. Uno et al.[15] observed a quick change of nss-$SO_4^{2-}$ concentrations in aerosol particles in the Kyushu District, located in the western part of Japan and closer to the Asian Continent, by taking aerosol samples hourly. They succeeded in reproducing the change of nss-$SO_4^{2-}$ concentrations by their elaborate transportation model, and concluded that the quick change in concentrations could be attributed to long-range transportation. This may be because the Hokuriku District is located farther from the Asian Continent than the Kyushu District and the plume of aerosol particles had a wider distribution. The monthly averaged nss-$SO_4^{2-}$ concentrations shown in Fig. 5 presented another aspect about the origin of the nss-$SO_4^{2-}$ in aerosol particles. In the figure, the averaged concentrations of winter season and other seasons were superimposed with horizontal bars. Because the typical air pressure pattern, which brings northwestern wind over Japan in the winter, i.e., high in the west and low in the east over Japan, is often seen from November to March, we defined this period as winter. Lower concentrations of nss-$SO_4^{2-}$ in aerosol particles were observed in winter rather than in other seasons. This seems to contradict the higher sulfate deposition in the winter.

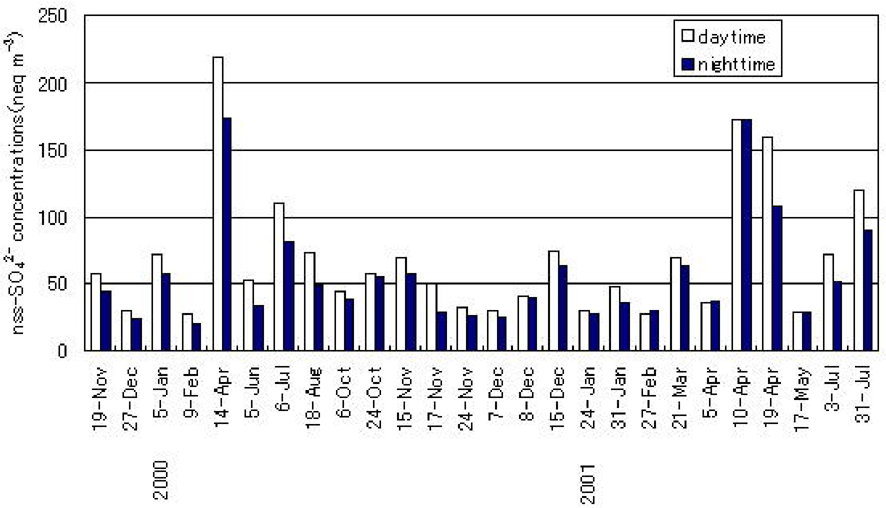

**FIGURE 4.** Atmospheric concentrations of nss-$SO_4^{2-}$ in aerosol particles in the daytime and nighttime.

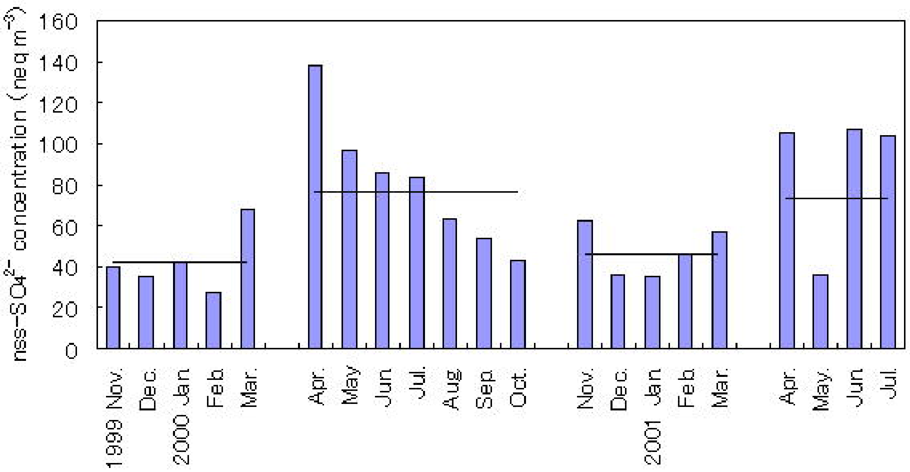

**FIGURE 5.** Monthly averaged concentration of nss-$SO_4^{2-}$ in aerosol particles. The horizontal bars denote the average concentration during each period.

### Washout Ratio

Atmospheric sulfur dioxide whose concentration decreased near 0 in the nighttime could not be attributed to the main cause of nss-$SO_4^{2-}$ observed in rain and snow since the concentrations in the precipitation did not differ much between daytime and nighttime. Neither could the lower concentration of nss-$SO_4^{2-}$ in aerosol particles observed in the winter be attributed to the elevated wet deposition. Therefore, it was presumed that the seasonal variation in washout efficiency of nss-$SO_4^{2-}$ in aerosol particles brought the higher deposition of nss-$SO_4^{2-}$ in the winter. We think the lower elevation of the cloud base could be one of the reasons of this phenomenon. In winter, the snowfall in this district is mainly brought by the northwestern anabatic wind as it rises over Japan Alps, located leeward of this district. Because plenty of humidity is supplied to the air mass as it traverses the Japan Sea before it reaches the Japan Alps to bring snowfall, a lower altitude of cloud base is often observed. It is likely that the clouds with a lower base could easily scavenge the air pollutants near the ground surface and that the effective washout of aerosol particles caused by the clouds with a lower base resulted in the lower concentration of nss-$SO_4^{2-}$ in aerosol particles and higher deposition in the winter in this district. Igawa et al. investigated the chemical composition in the fogwater collected at Oyama Mountain in Japan and reported that the lower cloud base resulted in the higher sulfate concentration in the fogwater[16].

As a result, a higher washout ratio, which is an indicator of scavenging efficiency[17,18], was observed in the winter.

The washout ratio W, is defined as

$$W = C\, \rho\, K^{-1}$$

where C (mg kg$^{-1}$) is the concentration of nss-$SO_4^{2-}$ in precipitation, ρ (kg m$^{-3}$) is the density of air, and K (mg m$^{-3}$) is the atmospheric concentration of nss-$SO_4^{2-}$ in aerosol particles during rain and snow. Fig. 6 shows the variation of washout ratios in winter and other seasons along with the intensity of precipitation that was averaged during the sampling period. Although the washout ratio is thought to depend highly on the intensity of precipitation[19], the regression lines indicated that the washout ratio in winter season was higher regardless of the intensity of precipitation.

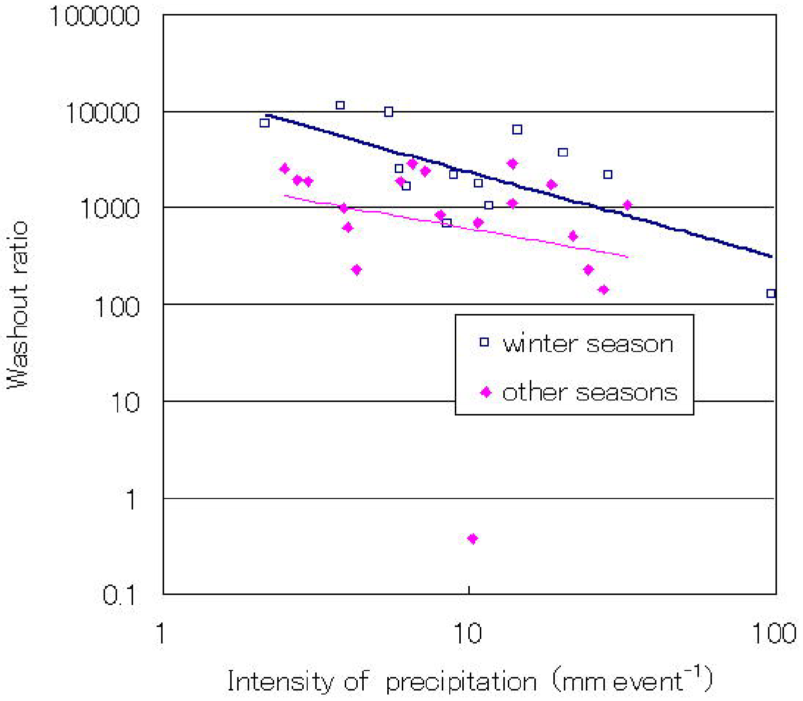

**FIGURE 6.** Washout ratios as a function of intensity of precipitation in winter and other seasons.

### CONCLUSIONS

Sulfur dioxide, which had a distinct diurnal pattern in concentration, originated in local emissions. Sulfur dioxide was found not to be essential for nss-$SO_4^{2-}$ in precipitation, because no diurnal pattern was found in the concentration of nss-$SO_4^{2-}$ in precipitation. Higher wet deposition of nss-$SO_4^{2-}$ was observed in the winter than in other seasons, while the concentration of nss-$SO_4^{2-}$ in aerosol particles was lower in the winter than in other seasons. This contradiction could be attributed to the lower cloud base, which brought the higher washout ratio in the winter. The nss-$SO_4^{2-}$ in aerosol particles, which had no diurnal pattern in its concentration, might result from long-range transportation.

### BIOSKETCH

**Tomonori Kawakami, Ph.D.**, is currently an Associate Professor of Environmental Systems Engineering at the College of Technology of Toyama Prefectural University. His research interests are acid rain, nitrate, nitrogen saturation, acidification of fresh water, atmospheric environment, aerosol, and Kosa (bai) event.
